# A Retrospective Age Analysis of the Ambulatory Oncology Patient Satisfaction Survey: Differences in Satisfaction across Dimensions of Person-Centred Care and Unmet Needs among Older Adults Receiving Cancer Treatment

**DOI:** 10.3390/curroncol31030113

**Published:** 2024-03-13

**Authors:** Fay J. Strohschein, Siwei Qi, Sandra Davidson, Claire Link, Linda Watson

**Affiliations:** 1 Faculty of Nursing, University of Calgary, Calgary, AB T2N 1N4, Canada; 2 Cancer Strategic Clinical Network, Alberta Health Services, Edmonton, AB T5J 3E4, Canada; 3 Applied Research & Patient Experience, Cancer Care Alberta, Alberta Health Services, Calgary, AB T2S 3C3, Canada

**Keywords:** Ambulatory Oncology Patient Satisfaction Survey, patient-reported experience measure, cancer care, age analysis, older adults, patient experience, patient satisfaction, aged, needs assessment, neoplasms

## Abstract

Over half of all new cancer cases in Alberta are diagnosed among people aged 65+ years, a group that encompasses vast variation. Patient-reported experience measures are routinely collected within Cancer Care Alberta; however, the specific consideration of the needs and concerns of older Albertans with cancer is lacking. In 2021, 2204 adults who had received treatment at a cancer centre in Alberta completed the Ambulatory Oncology Patient Satisfaction Survey (AOPSS). In this study, we explored the age differences in satisfaction across six dimensions of person-centred care and in the proportions of unmet needs across eight types of issues, with specific attention to older adults. Using three age groups (18–39, 40–64, 65+), only the physical comfort dimension showed significantly lower satisfaction among those aged 65+ years. Using five age groups (18–39, 40–64, 65–74, 75–84, 85+), significantly lower levels of satisfaction were found related to ‘physical comfort’ for those aged 65–74 and 75–84, ‘coordination and continuity of care’ for those aged 75–84 and 85+, and ‘information, communication, and education’ for those aged 85+. Therefore, grouping together all older adults aged 65+ years obscured lower levels of satisfaction with some dimensions of person-centred care among those aged 75–84 and 85+ years. Unmet needs generally increased with age for all types of issues, with significant differences across age groups for emotional, financial, social/family, and sexual health issues. The lower levels of satisfaction and higher proportions of unmet needs call for tailored interventions to promote optimal care experiences and outcomes among older adults receiving cancer care in Alberta and their families.

## 1. Introduction

In Alberta, Canada, 57% of new cancer cases were diagnosed among people aged 65+ years in 2021 [1]. Age-related health, functional, psychosocial, and existential changes [2] impact cancer care experiences, preferences, and outcomes [3,4,5]; however, little programmatic attention has been given to the unique concerns of this population in Alberta. Documented disparities among older adults with cancer, including over- and under-treatment [6,7], slower improvements in survival [8,9], unmet needs [10,11,12], and a lack of research [13,14,15], suggest that age-related changes may not be adequately addressed in cancer care. Given that the number of older Albertans has more than doubled in the past 20 years and is expected to nearly double again in the next 20 years [16], Cancer Care Alberta must strategically prepare to address older adults’ particular care needs.

Patient-reported experience measures (PREMs) provide insights into patients’ perceptions of their personal care experiences. They play a critical role in health system quality improvement through the production of knowledge to inform the development of health practices and policies that align with patient experiences and needs [17]. Specifically, PREMs can be used to address inequalities in care experiences, identifying groups of patients who report poorer experiences of care to prioritize initiatives that optimize experiences and outcomes [18,19].

The Ambulatory Oncology Patient Satisfaction Survey (AOPSS) [20,21] is a PREM used across many Canadian provinces to assess patients’ cancer care experiences. Since 2004, the AOPSS has been distributed every two years to people receiving cancer care in Alberta. Analysis has provided important insights to inform quality improvement initiatives and cancer care innovations in Alberta [22,23,24] and in other provinces [25,26,27]; however, little consideration has been given to the age-specific experiences of older adults with cancer.

In AOPSS analyses, adults aged 65+ are often grouped together, creating a single group that typically includes more than half of all respondents [21,22]. However, the life stage and experiences of a 65-year-old may differ greatly from those of a 75- or 85-year-old. Therefore, sociologists break down the older adult population into life-stage subgroups, often identified as the young–old (65–74 years), the middle–old (75–84 years), and the old–old (85+ years) [28]. Although age-related changes happen at different times and in different ways for different people [2], resulting in vast heterogeneity in health and functional status among older adults within each of these chronological groups [29], the consideration of the differences among these age groups begins to recognize the heterogeneity among older adults. Studies of patient satisfaction with cancer care in other jurisdictions that break down the older adult population into subgroups have shown lower levels of satisfaction in very old adults [18]. However, differences in healthcare context and available resources can have an important impact on satisfaction. Little is known about cancer care satisfaction among subgroups of older adults in the Canadian—and specifically the Alberta—context.

Therefore, the purpose of this study was to better understand the concerns and needs of older Albertans with cancer through a retrospective age analysis of the 2021 Alberta AOPSS. Specifically, we explored age differences in satisfaction across six dimensions of person-centred care and in the proportion of unmet needs across eight types of issues among adults receiving cancer care in Alberta, with particular attention to older adults. The findings may be used to inform the implementation of health system innovations that improve the experiences and outcomes of older people with cancer in Alberta and their families.

## 2. Materials and Methods

### 2.1. Design

We used a retrospective exploratory design to conduct a secondary age analysis of the 2021 AOPSS Alberta dataset.

### 2.2. Procedure

In Alberta, the AOPSS was distributed from February to May 2021. In February, potential respondents received a package in the mail containing an information sheet; a paper copy of the survey; a self-addressed, stamped return envelope; and a link to an online version of the survey with a unique patient identifier code. In March, a reminder was sent to those who had not yet returned the survey.

### 2.3. Respondents

A total of 4000 survey packages were mailed to patients with a cancer diagnosis who had received at least one systemic (intravenous or oral route) or radiation treatment at one of the 17 ambulatory cancer centres in Alberta in the previous 6 months. Some participants may have received both types of treatment. Eligible patients were identified from the Alberta Cancer Registry. A random sample of eligible patients was taken for the 2 metro cancer centres in Calgary and Edmonton. To ensure adequate representation of those living in smaller urban, rural, and remote areas, a census sample of patients was taken for the 4 regional and 11 community cancer centres.

### 2.4. Measure

The AOPSS was developed and nationally validated by the National Research Corporation (NRC) in 2003 [20]. After minor changes, the revised tool was again validated in 2012 [21]. Administered across many jurisdictions in Canada, the NRC managed the survey and maintained a national dashboard of results until 2023.

The AOPSS contains 97 questions [20,21,30]. Of these, 82 questions address experiences across the trajectory of cancer care. From these, the NRC identified 44 core questions to construct six validated dimensions of person-centred care, including (1) respect for patient preferences; (2) physical comfort; (3) access to care; (4) coordination and continuity of care; (5) information, communication, and education; and (6) emotional support [20,21]. The AOPSS also includes a question asking, ‘Did you get all of the help you wanted to cope with the following? (a) Practical issues (e.g., transportation, accommodation), (b) Financial issues (e.g., costs of treatments), (c) Social/family issues (e.g., worry about friends and family), (d) Emotional issues (e.g., fears and worries, sadness), (e) Spiritual issues (meaning/purpose of life, faith), (f) Informational issues (e.g., understanding your illness, talking with the healthcare team), (g) Physical issues (e.g., pain, fatigue), (h) Sexual health issues’ (No; Yes, somewhat; Yes, mostly; and Yes, definitely). In addition, a question about the overall rating of care asked, ‘Overall, how would you rate the quality of care at [cancer centre name] in the past 6 months?’ (Excellent, Very Good, Good, Fair, Poor) [21]. In the 2021 AOPSS, five Alberta-specific questions addressed experiences and satisfaction with virtual care, the goal of treatment, prognosis and advance care planning, and the involvement of the family physician. The survey ends with seven sociodemographic questions and an open-ended question for any other comments the respondent would like to make about their cancer care services.

Respondents chose to complete and return a paper copy of the survey or to complete the survey online. A contact phone number was provided to answer any survey-related questions.

In addition to the AOPSS survey data, the following associated cancer registry data were used: age (at survey distribution), tumor group, type of treatment received, cancer centre, and the first three digits of each respondent’s home postal code (forward sortation area, FSA).

### 2.5. Analysis

We selected the age groups for our analysis based on accepted definitions, ensuring that all groups included 50 or more respondents. In Alberta, young adults with cancer are defined as those aged 15–39 years [31] and older adults are typically defined as those aged 65+ years [32,33]. We used established sociological definitions to further categorize older adults as young–old (65–74 years), middle–old (75–84 years), and old–old (85+ years) [28].

The location data available were limited to the FSA for each respondent’s home address. With the assistance of a data and geospatial resources specialist, we used Geographic Information System (GIS) software, ArcGIS Pro version 2.9, to determine rurality by associating each FSA with the largest overlapping Alberta Health Services 2018 Official Local Geographic Area (LGA) and its corresponding classification on the Alberta Health Services Rural–Urban Continuum [34]. The following groupings were used for the rural–urban continuum classifications: ‘metro’ included metro centres (Edmonton, Calgary) and metro-influenced areas; ‘urban’ included only urban centres (Grand Prairie, Fort McMurray, Red Deer, Lethbridge, Medicine Hat); ‘rural and remote’ included moderately urban-influenced areas, large rural centres and surrounding areas, rural areas, and rural remote areas [34].

The sociodemographic, health, and clinical characteristics of the patients were analyzed using descriptive statistics. We tested for significant differences across age groups using Pearson’s chi-square tests for nominal variables and independent-samples Kruskal–Wallis tests for ordinal variables. For these tests, we used pairwise (test-by-test) deletion of respondents with missing data. For variables tested with Pearson chi-square tests, if the cells had an expected count of 5 or less, the response levels were combined or Monte Carlo estimates of exact *p*-values were used. For Pearson’s chi-square tests, we performed post hoc tests using *z* tests for independent proportions. For independent-samples Kruskal–Wallis tests, we performed post hoc tests using pairwise comparisons. In both cases, *p*-values were adjusted using the Bonferroni correction for multiple tests.

The primary outcome of interest was person-centred care, assessed using the six dimensions listed above. We treated the scores for each dimension as continuous variables. To investigate the impact of the patients’ age group on perceived person-centred care, we conducted a one-way multivariate analysis of variance (MANOVA). The MANOVA model allowed for a single statistical test across all six dimensions of person-centred care, reducing the risk of false positive results, which can occur when conducting separate tests for each dimension. For the MANOVA, we used listwise deletion of respondents with missing data on any dimension. The equality of covariance matrices was tested using Box’s test and the equality of error variances was tested using Levene’s test based on the median, which is more robust than the mean for skewed data [35]. If a significant main effect of the age group was observed, further post hoc tests were performed using Fisher’s least significant difference test to determine the specific differences for each dimension between age groups. The first one-way MANOVA investigated the impact of using a three-level age grouping (18–39, 40–64, and 65+) as the independent variable on the six person-centred dimension scores, which served as the dependent variables. The second one-way MANOVA investigated the impact of using a five-level age grouping (18–39, 40–64, 65–74. 75–84, and 85+) as the independent variable on the six person-centred dimension scores, which served as the dependent variables.

We used independent-samples Kruskal–Wallis tests to compare unmet needs and the overall rating of satisfaction with cancer centre care, which were constructed as ordinal variables using all response categories, across age groups. For these tests, we used pairwise (test-by-test) deletion of respondents with missing data. Post hoc pairwise comparisons were conducted when we found a significant difference across age groups, with *p*-values adjusted using the Bonferroni correction for multiple tests. 

Initial data cleaning, exploration, and figure creation were done using Microsoft Excel for Microsoft 365 MSO (Version 2302). For statistical tests, the data were exported to IBM SPSS Statistics (Version 25 or 27) for analysis, and a predetermined level of statistical significance was set at *p* < 0.05.

## 3. Results

Of the 4000 surveys distributed, 39 (1.0%) were undeliverable and 2204 were returned, giving a 55.6% response rate.

### 3.1. Sociodemographic, Health, and Clinical Characteristics

#### 3.1.1. Age Distribution of AOPSS Respondents

**Figure 1:**
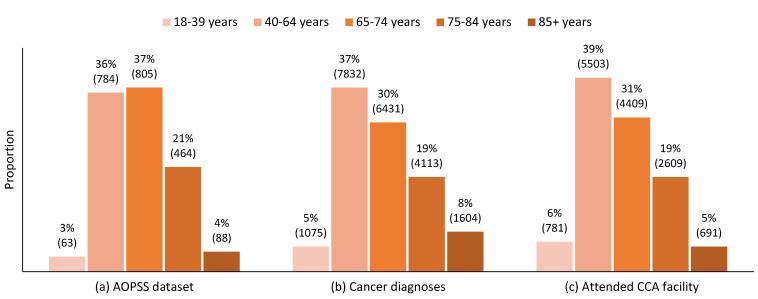
The 2021 comparative age distributions of (**a**) Ambulatory Oncology Patient Satisfaction Survey (AOPSS) respondents, (**b**) new cancer diagnoses in Alberta, and (**c**) people diagnosed with cancer that attended a Cancer Care Alberta facility. Note: n for each group is indicated in brackets.

The age distribution of the survey respondents (Figure 1a) reflects the age distribution of new cancer diagnoses in Alberta (Figure 1b) and of people with cancer who attended a Cancer Care Alberta facility (Figure 1c) in 2021, with a notable over-representation of those aged 65–74 years and under-representation of the youngest and oldest groups.

#### 3.1.2. Sociodemographic and Health Characteristics

There were significant differences across age groups for sex, education, the person who completed the survey, and self-rated health (Table 1). Post hoc pairwise comparisons between age groups are presented in Appendix A, Table A1. The oldest age group (85+ years) had the highest proportion of male respondents (*n* = 51, 58.0%) and of respondents with less than high school education (*n* = 35, 39.8%). Although not significantly different across age groups, the highest proportion of those living in rural and remote areas was among those aged 65–74 years (*n* = 404. 50.2%), closely followed by those aged 85+ years (*n* = 44, 50.0%) and 75–84 years (*n* = 227, 48.9%).

The proportion of surveys completed with help or by someone other than the patient in the 85+ age group (*n* = 36, 36.4%) was significantly higher than for all other age groups and that in the 75–84 age group (*n* = 82, 17.7%) was significantly higher than the 40–64 and 65–74 age groups (Table 1 and Table A1).

Generally, the proportion of those reporting excellent or very good health decreased with age, while the proportion of those reporting fair or poor health increased with age (Table 1). Specifically, those aged 85+ reported significantly poorer health than those aged 18–39, 40–64, and 65–74 years (Table A1). Notably, the proportion of those reporting good health remained relatively constant across age groups (Table 1).

#### 3.1.3. Clinical Characteristics

There were statistically significant differences across age groups with respect to the tumor site, time since diagnosis, type of cancer centre, involvement of the family doctor, treatment intent, and type of treatment(s) received (for IV and oral chemotherapy) (Table 2). Post hoc pairwise comparisons between age groups are presented in Appendix A, Table A2. In the younger age groups (18–39 years and 40–64 years), the highest proportion of respondents had breast cancers (*n* = 29, 46.0% and *n* = 248, 31.6%, respectively). In the older age groups (65–74, 75–84, and 85+ years), the highest proportion of respondents had hematological cancers and the proportion increased with age (*n* = 214, 26.6%; *n* = 153, 33.0%; and *n* = 39, 44.3%, respectively). This was also reflected in the post hoc comparisons (Table A2). Among the oldest respondents (85+ years), most had been living with cancer for two or more years (*n* = 58, 65.9%), whereas, among the youngest respondents (18–39 years), most had received their diagnosis within the past year (*n* = 35, 55.6%). Accordingly, those aged 18–39 years had a significantly shorter time since diagnosis than all other age groups and those aged 85+ years had a significantly longer time since diagnosis than all other age groups (Table A2). 

The young adult group (18–39 years) had the highest proportion of respondents receiving care at a metropolitan cancer centre (Table 2); however, the post hoc comparisons did not show any significant differences between age groups (Table A2). The proportions of those receiving care at regional cancer centres were, however, significantly higher among those aged 65–74 years (*n* = 246, 30.6%) and 75–84 years (*n* = 154, 33.2%) than those aged 40–64 years (*n* = 181, 23.1%) (Table 1 and Table A1). The highest proportion of those receiving care at community (rural) cancer centres was in the middle-aged group (40–64 years; *n* = 122, 15.6%); however, the post hoc comparisons again did not show any significant differences across age groups (Table A2).

There was an increase with age in the proportion of respondents who reported that their family doctor was very involved and the proportion of respondents unsure about their family doctor’s involvement also increased with age (Table 2). However, the only significant differences were in those aged 40–64 years having significantly less family doctor involvement than those aged 75–84 and 85+ years (Table A2).The proportion of respondents reporting a treatment intent of control, rather than cure, also increased with age, with almost two thirds (*n* = 54, 61.4%) of those in the oldest group (85+ years) reporting control as the treatment intent (Table 2), a significantly greater proportion than all other age groups (Table A2). Interestingly, the oldest age group also showed the highest proportion of respondents who were unsure about their treatment intent (*n* = 6, 6.8%) or left the question unanswered (*n* = 12, 13.6%). 

### 3.2. Dimensions of Person-Centred Care

#### 3.2.1. Three-Level Age Grouping

**Figure 2:**
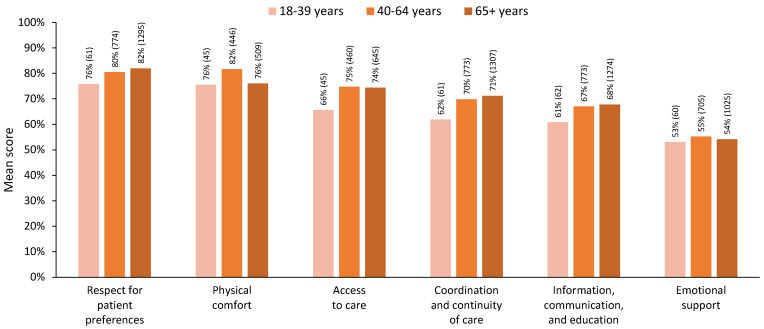
Mean scores for each dimension of person-centred care by age group, using three age groups. Note: All respondents with valid dimension scores are included in this figure; *n* for each group is indicated in brackets.

For the six dimensions of person-centred care, when three age groups were used, the differences across age groups primarily pointed towards lower satisfaction for young adults (18–39 years, Figure 2). For the MANOVA, we included 781 respondents who had complete data across all dimensions (18–39 years, n = 39; 40–64 years, n = 361; 65+ years, n = 381). The MANOVA results showed a statistically significant difference in perceived person-centred care based on patients’ three-level age groups, Pillai’s trace = 0.032, F(12, 1548) = 2.104, *p* = 0.014. The assumption of equality of covariance matrices in MANOVA was not violated (Box’s M = 51.3, *p* = 0.198). Although Levene’s median-based test for equal variances suggested that the assumption of homogeneity of variances was met in five dimensions, it was violated for the ‘physical comfort’ dimension, F(2, 778) = 5.50, *p* = 0.004. Nevertheless, we proceeded with the MANOVA, considering its robustness against slight violations of this assumption [36] and reporting Pillai’s trace, which is the most robust test statistic when assumptions are violated [36,37].

Post hoc comparisons showed significantly lower levels of satisfaction for younger adults in the ‘access to care’ and ‘coordination and continuity of care’ dimensions (*p* < 0.05). Satisfaction for older adults aged 65+ was similar to or higher than satisfaction in the younger age groups for all dimensions except physical comfort, in which satisfaction for those 65+ years was significantly lower than for those aged 40–64 years (*p* < 0.05). Detailed statistics are presented in Table 3.

#### 3.2.2. Five-Level Age Grouping

**Figure 3:**
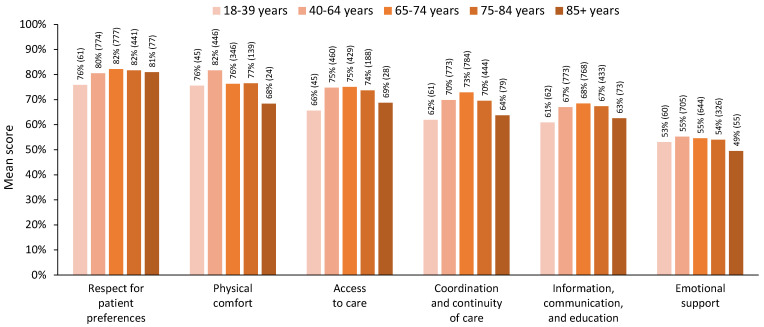
Mean score for each dimension of person-centred care scores by age group, using five age groups. Note: All respondents with valid dimension scores are included in this figure; n for each group is indicated in brackets.

When five age groups were used to explore the age differences among the dimensions of person-centred care, a different story emerged. Decreasing patterns of satisfaction for older adults aged 75–84 years were evident across most dimensions, and those aged 85+ years showed levels of satisfaction lower than those aged 65–74 years on all dimensions of person-centred care (Figure 3). For the MANOVA, we included 781 respondents who had complete data across all dimensions (18–39 years, n = 39; 40–64 years, n = 361; 65–74 years, n = 264; 75–84 years, n = 105; 85+ years, n = 12). The MANOVA results showed a statistically significant difference in perceived person-centred care based on a patient’s five-level age group, Pillai’s trace = 0.059, F(24, 3096) = 1.94, *p* = 0.004. The assumption of equality of covariance matrices in the MANOVA was met (Box’s M = 95.2, *p* = 0.373). Although Levene’s median-based test for equal variances suggested that the assumption of homogeneity of variances was met in four dimensions, it was violated for the ‘physical comfort’ dimension, F(4, 776) = 2.98 *p* = 0.019, and the ‘emotional support’ dimension, F(4, 776) = 2.78, *p* = 0.026. Nevertheless, we proceeded with the MANOVA, considering its robustness against slight violations of this assumption [36] and reporting Pillai’s trace, which is the most robust test statistic when assumptions are violated [36,37].

Post hoc comparisons showed significantly lower satisfaction for those aged 75–84 and 85+ years on several dimensions. Specifically, older adults aged 75–84 years and 85+ years showed significantly lower satisfaction in the ‘coordination and continuity of care’ dimension than adults aged 40–64 years (*p* < 0.05). Older adults aged 85+ years also showed significantly lower satisfaction in this dimension than adults aged 65–74 years (*p* < 0.05). Moreover, adults aged 85+ years showed significantly lower satisfaction than those aged 40–64, 65–74, and 75–84 years on the ‘information, communication, and education’ dimension (*p* < 0.05). Consistent with the three-level age group analysis, older adults aged 65–74 years and 75–84 years showed significantly lower levels of satisfaction on the ‘physical comfort’ dimension than adults aged 40–64 (*p* < 0.05). Adults aged 85+ had the lowest level of satisfaction on the ‘physical comfort’ dimension; however, this did not show significance in the post hoc tests due to the smaller sample size. Detailed statistics are presented in Table 4.

### 3.3. Unmet Needs

**Figure 4:**
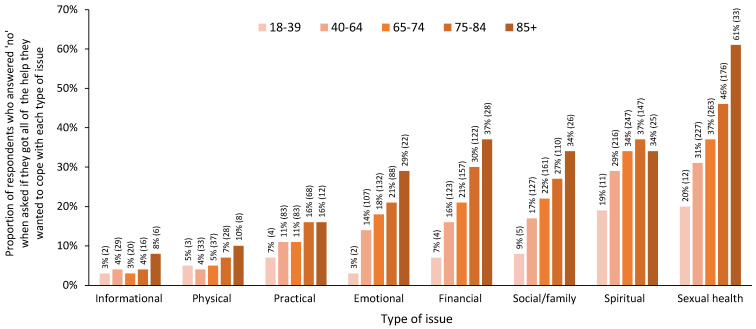
Proportion of respondents reporting that they did not receive the help that they wanted across eight types of issues by age group, using five age groups. Note: For simplicity, only the proportion of ‘no’ responses for each age group is represented in this figure; *n* for each group is indicated in brackets. Detailed statistics for significance testing, including all response categories, are presented in Table 5.

When respondents were asked if they had received the help that they wanted related to eight types of issues, the proportion of respondents who answered ‘no’ generally increased with age across all types of issues (Figure 4). The highest proportion of unmet needs was found among those aged 75–84 and/or 85+ years across all types of issues (Figure 4). The differences in responses across age groups were significant for emotional, financial, social/family, and sexual health issues (*p* < 0.05). The differences across age groups for practical and spiritual issues were also nearing statistical significance (*p* = 0.099 and *p* = 0.069, respectively). Detailed statistics for the significance testing, including all response categories, and pairwise comparisons are reported in Table 5.

### 3.4. Overall Ratings of Care across Five Age Groups

There was a statistically significant difference across age groups when respondents were asked about their overall quality of care, H(4) = 12.84, *p* = 0.012. The post hoc pairwise comparisons demonstrated that those aged 85+ years reported significantly lower satisfaction with their overall quality of care than those aged 65–74 years (*p* < 0.05). Detailed statistics are presented in Table 6.

## 4. Discussion

In this study, we conducted an age analysis of the 2021 Alberta AOPSS survey data. Our primary outcome of interest was satisfaction across six dimensions of person-centred care. When we used three age groups, those aged 65+ years showed levels of satisfaction approximately equal to or greater than those aged 18–39 years and 40–64 years on all dimensions of person-centred care, except for physical comfort, for which satisfaction was significantly lower for those aged 65+ years than those aged 40–64 years. However, when we used five age groups, dividing the older adults into three groups, a decreasing pattern of satisfaction for those aged 75–84 years was evident on most dimensions, and those aged 85+ years showed levels of satisfaction lower than those aged 65–74 years on all dimensions of person-centred care.

The MANOVA results showed a statistically significant difference across age groups for both analyses. However, the post hoc comparisons with three age groups pointed towards lower levels of satisfaction primarily in the 18–39 years age group, except for the ‘physical comfort’ dimension, which was significantly lower in those aged 65+ years. With the five age groups, the post hoc analysis confirmed the significantly lower levels of satisfaction for older adults on the ‘physical comfort’ dimension, specifically among those aged 65–74 and 75–84. In addition, this analysis showed significantly lower levels of satisfaction among those aged 75+ on the ‘coordination and continuity of care’ dimension and for those aged 85+ on the ‘information, communication, and education’ dimension.

This analysis of a large sample of people receiving cancer care in Alberta highlights how using a single group for all older adults aged 65+ years can obscure the lower levels of satisfaction among those aged 75–84 and 85+ years, particularly on the ‘coordination and continuity of care’ and ‘information, communication, and education’ dimensions. Older adults are a vastly heterogeneous group. Although chronological age is only a rough proxy for the variation in health and functional status that occurs among older adults [29], dividing older adults into multiple age groups begins to acknowledge the variation in patient-reported experiences.

The dimensions that showed lower satisfaction among older adults are consistent with the existing understanding of age-related concerns. Multimorbidity increases with age, affecting only 13.3% of Canadians aged 20–64 but 32.8% of Canadians aged 65–74, 42.7% of Canadians aged 75–84, and 47.7% of Canadians aged 85+ years [38]. Multimorbidity may contribute to greater physical discomfort during cancer care, affecting the choice and completion of treatment [39]. In addition, the management of multiple morbidities calls for additional coordination of care among multiple medical specialists, primary care providers, and allied healthcare providers. Age-related health, functional, and social changes may also interact with cancer-related changes and require active coordination among cancer care providers and community health or social care services during and after cancer treatment. Furthermore, shifting values related to quality and quantity of life [5] and a lack of research to inform treatment decisions among older adults with cancer [13] can contribute to greater complexity in the treatment decision-making process, calling for greater coordination, as well as intentional information sharing and communication, among healthcare providers, patients, and families/caregivers. Communication challenges among older adults with cancer and their care providers are not new and may be impacted by age-related sensory, cognitive, and functional changes that impact interactions; the involvement of families/caregivers; and/or ageist attitudes among both care providers and patients [40,41]. Decreased satisfaction among older adults on these dimensions of person-centred care is critical given the potential impact on health outcomes.

In addition, the proportion of respondents who did not receive the help that they wanted generally increased with age across all types of issues. The difference across age groups was statistically significant for emotional, financial, social/family, and sexual health issues, and it neared statistical significance for practical and spiritual issues. This finding is consistent with previous studies that have identified unmet needs among older adults diagnosed with cancer [10,42], among older adults undergoing active cancer treatment [11,43], and among older cancer survivors [12]. The types of unmet needs highlighted, however, vary widely across studies, including medical issues [10]; informational issues [10,11,42,43,44]; practical issues, such as transportation or insurance [12,42]; financial issues [12,44]; psychological issues [11,12]; physical issues [11,12]; relational issues [12]; communication issues [42,43]; spiritual issues [44]; and issues relating to coordination among care providers, including primary care providers [42]. The differences in unmet needs across studies may reflect differences in the measures used, as well as variations in the health system context, specifically related to the available services and resources.

Notably, in a previous Canadian study of cancer survivors, researchers also found a high number of older adults expressing concern about sexual issues, with a high proportion reporting that they did not receive the help that they wanted [12], echoing the high proportion of unmet sexual health issues among older adults in this study. Communication by healthcare providers about sexual side effects has been found to decrease as patient age increases [41]. The use of sexual health assessment tools, and an awareness of the potential impact of cancer and cancer treatment on sexual health, may help to address the unmet needs related to sexual health among older adults with cancer [45]. 

Finally, the overall rating of the care at cancer centres also showed significant differences across age groups. The pairwise comparisons pointed towards lower levels of satisfaction with the quality of care among those aged 85+ years. Across all these analyses, it is important to note the overall pattern of lower satisfaction and unmet needs among those aged 85+ years. A strength of this study was having a sufficient sample size to detect significant differences for this group. In Alberta, in 2021, the number of cancer diagnoses among those aged 85+ years was about 50% higher than that among those aged 18–39 years, with both groups comprising similar proportions of those attending a cancer centre, 6% for those aged 18–39 years and 5% for those aged 85+ years (Figure 1). Current estimates suggest that the number of Canadians aged 85+ with cancer will more than double (increase by 130%) in the next 20 years [46]. In Alberta, previous AOPSS results have informed the development of programs and services tailored to the needs and concerns of young adults with cancer; the results of this age analysis clearly highlight the need for services and resources tailored to the needs and concerns of older adults with cancer and their families/caregivers.

### 4.1. Implications

Insights from this age analysis can inform the development of services and resources tailored to support older adults with cancer and their families, highlighting which groups to target with various interventions. Interventions and services addressing physical comfort should target older adults aged 65+ years; those addressing coordination and continuity of care would most benefit those aged 75+ years; and tailored information, communication, and education would most benefit those aged 85+ years. Resources to address unmet needs, particularly those related to emotional, financial, social/family, and sexual health issues, should be considered for all older adults receiving cancer care in Alberta. Geriatric assessment and management (GAM) and patient navigation are key interventions to address these areas of concern.

GAM is an effective approach to understanding variation, addressing age-related concerns, and improving outcomes in the care of older adults with cancer [47]. Geriatric assessment is the most commonly reported supportive intervention for older people having cancer treatment [48]. In the American Society of Clinical Oncology guidelines, experts recommend GAM for patients aged 65+ with identified vulnerabilities, to inform cancer treatment decision making and supportive interventions to optimize treatment outcomes [49]. Randomized controlled trials have demonstrated that, among older adults receiving cancer treatment, GAM can reduce toxicity and complications; promote treatment completion; improve quality of life and physical function/mobility; increase age-related conversations among oncologists and patients; and improve communication satisfaction for patients and families/caregivers [47,49,50]. Therefore, GAM holds evidence-based potential for positive impacts in at least two of the dimensions of person-centred care that showed lower levels of satisfaction among older adults with cancer in Alberta.

Navigation is supported by strong evidence for improvements in patient satisfaction with care and quality of life, with emerging evidence for improved communication [51]. Specifically, in Canada, patients treated for cancer who were assigned a nurse navigator reported higher satisfaction across all dimensions of person-centred care on the AOPSS [27]. Among older adults with cancer specifically, a systematic review of navigation also found a positive impact on satisfaction [52]. Within Cancer Care Alberta, the cancer patient navigator role was designed to address concerns related to continuity of care, including informational, management, and relational continuity [53]. New models of cancer care navigation, including generalist navigators in rural settings, and population-specific navigators for Indigenous persons and young adults, have been successfully implemented in Alberta, decreasing emergency visits and hospital admissions and increasing positive care experiences [53,54]. Therefore, to address the lower levels of satisfaction among older adults identified in this study, particularly with respect to the coordination and continuity of care, opportunities exist to educate generalist navigators about best practices in geriatric oncology and to develop a population-specific navigator for older adults with cancer.

The clear involvement of families/caregivers in completing the survey among older adults with cancer highlights the need to include and address family/caregiver concerns in interventions for older adults with cancer [48]. GAM and navigation interventions also show promise for family/caregiver communication and support [50,51].

### 4.2. Future Directions

As the AOPSS is a bi-annual survey in Alberta, future analyses may consider longitudinal changes over time related to age differences in satisfaction, supporting the evaluation of interventions addressing age-related concerns. In this study, we used univariate analyses to explore the patterns and significant differences in satisfaction across dimensions of person-centred care, unmet needs, and the overall rating of cancer centre care across age groups. Future research could incorporate multivariate analyses to explore and provide a greater understanding of these relationships. However, given that health records often contain limited information concerning other sociodemographic characteristics and age is readily available, age may remain a valuable proxy to identify those requiring additional support.

The respondents for this survey were sampled from people who had received systemic or radiation treatment at a cancer centre in Alberta within the 6 months prior to survey distribution. Among adults aged 75+ years, and particularly among those aged 85+ years, those receiving systemic or radiation treatment may be an increasingly select sub-group of those who have been diagnosed with cancer. To fully understand care satisfaction and unmet needs among older adults with cancer, future research may seek to also understand the experiences of those not receiving active treatment, as well as those with suspected or clinical diagnoses for whom further diagnostic investigations are not pursued, providing a more comprehensive understanding of the supportive services and resources needed.

### 4.3. Limitations

Significantly lower levels of satisfaction were identified among the youngest (18–39 years) and oldest (85+ years) age groups. However, due to missing data and smaller sample sizes limiting the power to detect significant differences for these groups, we chose to use a more liberal and powerful post hoc test for the MANOVA—Fisher’s least significant difference test. This test is not typically recommended because it does not adjust the significance for multiple comparisons, raising the risk of a Type I error [55]. It does, however, decrease the risk of Type II errors, giving a sense of where there may be significant results if more conservative tests were used with larger sample sizes. In addition, although the proportion of unmet needs for several types of issues was highest for those aged 85+ years, we did not find significant differences for this group using the more conservative Bonferroni test for post hoc analysis. Therefore, approaches to increase the number of responses for the oldest and youngest groups and reduce missing data in future studies would strengthen the power and ability to use more conservative statistical analyses.

The distribution of the 2021 AOPSS survey coincided with the third wave of COVID-19 in Alberta [56]. During this time, many cancer care visits were conducted virtually, in-person supportive care activities were limited, and the presence of families/caregivers was restricted. Lower satisfaction with cancer care was noted in Alberta during the COVID-19 pandemic [57]; however, susceptibility to stress for cancer patients during the COVID-19 pandemic was not associated with age [58]. Therefore, it would not be reasonable to attribute the age differences in satisfaction found in this study to the COVID-19 pandemic alone. Age analysis of future patient-reported experience data collected after the COVID-19 pandemic will lead to a greater understanding of the ongoing age differences in care satisfaction.

The dataset used for this retrospective analysis did not include information about the health or functional status of respondents beyond self-rated health. Given the vast variation among older adults, challenges related to multimorbidity, activities of daily living, cognitive status, or mood may impact satisfaction with care and unmet needs [10,18,59,60]; however, data related to these domains are currently limited for older adults with cancer in Alberta. The greater integration of GAM into cancer care could provide opportunities to explore the relationships between care satisfaction and domains of geriatric concern, further informing targeted interventions to strengthen care experiences and outcomes.

Patients who experience sensory or cognitive deficits, have lower levels of education, lack the active involvement of their family/caregivers, or have higher levels of physical discomfort or fatigue may also be less able or willing to complete the lengthy AOPSS. In this study, many of these characteristics increased with age. Among older adults, we saw a higher proportion of surveys completed by, or with the help of, someone else and of missing data. Therefore, the respondents who chose, and were able, to complete and return the survey may have been different from those who were unable or chose not to do so, particularly among older adults. In future studies, the greater integration of interviews and/or telephone survey completion may facilitate the involvement of those facing barriers to survey completion [61], strengthening the representativeness of the results and increasing the responses.

As noted, among older adults, there was a higher proportion of AOPSS completed by, or with the help of, someone else. Therefore, the responses in the older age groups may reflect a greater proportion of family/caregiver perspectives, in addition to patient perspectives. Previous studies have found lower levels of satisfaction among families/caregivers as compared to patients in cancer care [62]. These family/caregiver perspectives, however, are also critical in informing quality improvements [63], suggesting a need for further research that considers both patient and family/caregiver satisfaction.

## 5. Conclusions

Grouping together all older adults aged 65+ years when analyzing data from patient experience measures can obscure the lower levels of satisfaction among those aged 75+ and 85+ years, resulting in important and nuanced age-related concerns in these older age groups being overlooked. The significantly lower levels of satisfaction among older adults in the dimensions of ‘physical comfort’, ‘coordination and continuity of care’, and ‘information, communication, and education’, as well as increasing unmet needs, significant for emotional, financial, social/family, and sexual health issues, highlight the need for programmatic attention, with tailored services and resources, to address the needs and concerns of older adults with cancer and their families in Alberta.

## Figures and Tables

**Table 1:** Distribution of sociodemographic and health characteristics by age group.

	Age Group (Years)					*p*
	18–39	40–64	65–74	75–84	85+	
**Sex ^1^**						<0.001
Female	44 (69.8%)	488 (62.2%)	401 (49.8%)	242 (52.2%)	37 (42.0%)	
Male	19 (30.2%)	296 (37.8%)	404 (50.2%)	222 (47.8%)	51 (58.0%)	
**Education ^2^**						<0.001
Less than high school	5 (7.9%)	82 (10.5%)	136 (16.9%)	138 (29.7%)	35 (39.8%)	
High school/college	29 (46.0%)	476 (60.7%)	447 (55.5%)	203 (43.8%)	34 (38.6%)	
University	28 (44.4%)	198 (25.3%)	168 (20.9%)	86 (18.5%)	8 (9.1%)	
Missing values ^3^	1 (1.6%)	28 (3.6%)	54 (6.7%)	37 (8.0%)	11 (12.5%)	
**Rurality ^1,4^**						0.365
Metro	33 (52.4%)	373 (47.6%)	334 (41.5%)	201 (43.3%)	37 (42.0%)	
Urban	4 (6.3%)	64 (8.2%)	67 (8.3%)	36 (7.8%)	7 (8.0%)	
Rural and remote	26 (41.3%)	346 (44.1%)	404 (50.2%)	227 (48.9%)	44 (50.0%)	
Missing values ^3^	0 (0.0%)	1 (0.1%)	0 (0.0%)	0 (0.0%)	0 (0.0%)	
**Who completed survey ^1^**						<0.001
Patient	56 (88.9%)	696 (88.8%)	686 (85.2%)	356 (76.7%)	47 (53.4%)	
Patient with help/someone else	6 (9.5%)	73 (9.3%)	88 (10.9%)	82 (17.7%)	32 (36.4%)	
Missing values ^3^	1 (1.6%)	15 (1.9%)	31 (3.9%)	26 (5.6%)	9 (10.2%)	
**Self-rated health ^2^**						<0.001
Excellent or very good	30 (47.6%)	281 (35.8%)	263 (32.7%)	139 (30.0%)	15 (17.0%)	
Good	23 (36.5%)	315 (40.2%)	337 (41.9%)	190 (40.9%)	36 (40.9%)	
Fair or poor	7 (11.1%)	167 (21.3%)	175 (21.7%)	118 (25.4%)	31 (35.2%)	
Missing values ^3^	3 (4.8%)	21 (2.7%)	30 (3.7%)	17 (3.7%)	6 (6.8%)	

^1^ For nominal variables, the significance of differences across age groups was calculated using Pearson chi-square tests; ^2^ for ordinal variables, the significance of differences across age groups was calculated using independent-samples Kruskal–Wallis tests; ^3^ testing for significant differences across age groups did not include missing values; ^4^ rurality was based on the first three digits of the respondent’s postal code—please see Section 2.5 for details.

**Table 2:** Distribution of clinical characteristics by age group.

	Age Group (Years)					*p*
	18–39	40–64	65–74	75–84	85+	
**Tumor site ^1^**						<0.001
Hematological	15 (23.8%)	160 (20.4%)	214 (26.6%)	153 (33.0%)	39 (44.3%)	
Breast	29 (46.0%)	248 (31.6%)	139 (17.3%)	68 (14.7%)	8 (9.1%)	
Gastrointestinal	6 (9.5%)	124 (15.8%)	125 (15.5%)	63 (13.6%)	4 (4.5%)	
Genitourinary	0 (0.0%)	56 (7.1%)	127 (15.8%)	69 (14.9%)	11 (12.5%)	
Intrathoracic	0 (0.0%)	67 (8.5%)	98 (12.2%)	59 (12.7%)	14 (15.9%)	
Gynecological	1 (1.6%)	44 (5.6%)	45 (5.6%)	24 (5.2%)	1 (1.1%)	
Skin ^2^	2 (3.2%)	25 (3.2%)	25 (3.1%)	11(2.4%)	8 (9.1%)	
Head and neck	1 (1.6%)	28 (3.6%)	14 (1.7%)	5 (1.1%)	1 (1.1%)	
Central nervous system	6 (9.5%)	18 (2.3%)	6 (0.7%)	3 (0.6%)	0 (0.0%)	
Other ^3^	3 (4.8%)	14 (1.8%)	12 (1.5%)	9 (1.9%)	2 (2.3%)	
**Time since diagnosis ^4^**						<0.001
Less than 6 months	9 (14.3%)	69 (8.8%)	54 (6.7%)	30 (6.5%)	1 (1.1%)	
6 months to 1 year	26 (41.3%)	275 (35.1%)	250 (31.1%)	143 (30.8%)	18 (20.5%)	
1 to 2 years	23 (36.5%)	178 (22.7%)	175 (21.7%)	67 (14.4%)	11 (12.5%)	
2 to 5 years	1 (1.6%)	131 (16.7%)	173 (21.5%)	109 (23.5%)	29 (33.0%)	
More than 5 years	4 (6.3%)	131 (16.7%)	153 (19.0%)	115 (24.8%)	29 (33.0%)	
**Type of cancer centre where care was received ^1^**						0.001
Metropolitan ^5^	45 (71.4%)	481 (61.4%)	448 (55.7%)	249 (53.7%)	50 (56.8%)	
Regional	14 (22.2%)	181 (23.1%)	246 (30.6%)	154 (33.2%)	29 (33.0%)	
Community (rural)	4 (6.3%)	122 (15.6%)	111 (13.8%)	61 (13.1%)	9 (10.2%)	
**Involvement of family doctor in care while on cancer treatment ^4^**						<0.001
Very involved	10 (15.9%)	163 (20.8%)	188 (23.4%)	120 (25.9%)	25 (28.4%)	
Somewhat involved	18 (28.6%)	226 (28.8%)	245 (30.4%)	144 (31.0%)	23 (26.1%)	
Not very involved	13 (20.6%)	172 (21.9%)	170 (21.1%)	90 (19.4%)	18 (20.5%)	
Not at all involved	14 (22.2%)	151 (19.3%)	121 (15.0%)	63 (13.6%)	5 (5.7%)	
Do not have ^6^	6 (9.5%)	22 (2.8%)	10 (1.2%)	2 (0.4%)	1 (1.1%)	
Unsure ^6^	1 (1.6%)	24 (3.1%)	34 (4.2%)	18 (3.9%)	5 (5.7%)	
Missing values ^7^	1 (1.6%)	26 (3.3%)	37 (4.6%)	27 (5.8%)	11 (12.5%)	
**Treatment intent ^1^**						<0.001
Cure	46 (73.0%)	382 (48.7%)	348 (43.2%)	184 (39.7%)	16 (18.2%)	
Control	15 (23.8%)	335 (42.7%)	381 (47.3%)	233 (50.2%)	54 (61.4%)	
Unsure	0 (0%)	16 (2.0%)	16 (2.0%)	12 (2.6%)	6 (6.8%)	
Missing values ^7^	2 (3.2%)	51 (6.5%)	60 (7.5%)	35 (7.5%)	12 (13.6%)	
**Type of cancer treatment(s) received (not mutually exclusive) ^1,8^**						
IV chemotherapy	41 (65.1%)	505 (64.4%)	467 (58.0%)	228 (49.1%)	28 (31.8%)	<0.001
Oral chemotherapy	16 (25.4%)	260 (33.2%)	282 (35.0%)	179 (38.6%)	44 (50.0%)	0.005
Radiation therapy	24 (38.1%)	248 (31.6%)	231 (28.7%)	124 (26.7%)	28 (31.8%)	0.194

^1^ For nominal variables, the significance of differences across age groups was calculated using Pearson chi-square tests; ^2^ skin includes melanoma and non-melanoma skin cancers; ^3^ other tumor sites include those represented in 25 or less respondents, including sarcoma, endocrine cancers, and other malignancies not specified; ^4^ for ordinal variables, the significance of differences across age groups was calculated using independent-samples Kruskal–Wallis tests; ^5^ metropolitan cancer centres are the largest cancer centres, located in Edmonton and Calgary; ^6^ for family doctor involvement, ‘do not have’ and ‘unsure’ were considered not applicable responses and were not included in the significance testing across age groups; ^7^ testing for significant differences across age groups did not include missing values; ^8^ based on tumor registry data—some respondents received more than one type of treatment.

**Table 3:** MANOVA results for dimensions of person-centred care using three age groups.

Dimensions of Person-Centred Care	*F* (2, 778)	*p*	Post Hoc Comparison ^1^	*p* ^2^
Respect for patient preferences	1.221	0.295	–	
Physical comfort	5.498	0.004	40–64 > 65+	0.001
Access to care	3.009	0.05	18–39 < 40–64	0.015
			18–39 < 65+	0.037
Coordination and continuity of care	3.203	0.041	18–39 < 40–64	0.019
Information, communication, and education	2.077	0.126	–	
Emotional support	0.727	0.483	–	

^1^ Only results of significant post hoc comparison are presented; ^2^ calculated using Fisher’s least significant difference test.

**Table 4:** MANOVA results for dimensions of person-centred care using five age groups.

Dimensions of Person-Centred Care	*F* (4, 776)	*p*	Post Hoc Comparisons ^1^	*p ^2^*
Respect for patient preferences	1.492	0.203	–	
Physical comfort	2.978	0.019	40–64 > 65–74	0.009
			40–64 > 75–84	0.011
Access to care	1.728	0.142	–	
Coordination and continuity of care	3.683	0.006	18–39 < 40–64	0.018
			18–39 < 65–74	0.031
			40–64 > 75–84	0.037
			40–64 > 85+	0.011
			65–74 > 85+	0.015
Information, communication, and education	2.482	0.043	40–64 > 85+	0.012
			65–74 > 85+	0.026
			75–84 > 85 +	0.017
Emotional support	0.397	0.811	–	

^1^ Only results of significant post hoc comparisons are presented; ^2^ calculated using Fisher’s least significant difference test.

**Table 5:** Independent-samples Kruskal–Wallis test results for receiving the help that respondents wanted across eight types of issues by age group, using five age groups.

Type of Issues	Total N	H(4)	*p*	Post Hoc Comparisons ^1^	Adj *p* ^2^
Informational	2116	7.022	0.135	–	–
Physical	2104	7.434	0.115	–	–
Practical	2060	7.8	0.099	–	–
Emotional	2070	18.065	0.001	40–64 > 75–84	0.009
Financial	2026	17.666	0.001	18–39 > 75–84	0.017
				40–64 > 75–84	0.02
Social/Family	2043	16.302	0.003	40–64 > 75–84	0.003
Spiritual	1990	8.707	0.069	–	–
Sexual Health	1941	18.65	<0.001	40–64 > 75–84	0.008

Note: All response categories were included in the significance testing presented in this table. ^1^ Only results of significant post hoc comparisons are presented; ^2^ significance values have been adjusted by the Bonferroni correction for multiple tests.

**Table 6:** Independent-samples Kruskal–Wallis test results and mean ranks for overall ratings of care by age group, using five age groups.

Question	Total N	H(4)	*p*	Age Group (Years)	n	Mean Rank	Post Hoc	Adj *p* ^2^
							Comparisons ^1^	
Overall, how would you rate the quality of care at [cancer care centre] in the past 6 months?	2124	12.842	0.012	18–39	61	987.17	65–74 > 85+	0.017
				40–64	773	1069.38		
				65–74	767	1091.24		
				75–84	444	1039.08		
				85+	79	905.96		

^1^ Only results of significant post hoc comparisons are presented; ^2^ significance values have been adjusted by the Bonferroni correction for multiple tests.

## Data Availability

Restrictions apply to the availability of these data, according to the Alberta Health Information Act (https://www.alberta.ca/health-information-act) and associated regulations. Data were obtained from Alberta Health Services and are only available with appropriate ethical and operational approval.

## Appendix A

This appendix contains tables with the results of the post hoc comparisons between age groups for sociodemographic and health characteristics (Table A1), which complement the results in Table 1, and clinical characteristics (Table A2), which complement Table 2. Please see Section 2.5 for a description of the analyses.

**Table A1:** Post hoc comparisons between age groups for sociodemographic and health characteristics showing a significant difference across age groups.

	Post Hoc Comparisons	Adj *p ^3^*
**Sex ^1^**		
Female	18–39 > 65–74	0.022
	18–39 > 85+	0.007
	40–64 > 65–74	<0.001
	40–64 > 75–84	0.005
	40–64 > 85+	0.002
Male	18–39 < 65–74	0.022
	18–39 < 85+	0.007
	40–64 < 65–74	<0.001
	40–64 < 75–84	0.005
	40–64 < 85+	0.002
**Education ^2^**		
	18–39 > 65–74	0.001
	18–39 > 75–84	<0.001
	18–39 > 85+	<0.001
	40–64 > 65–74	0.011
	40–64 > 75–84	<0.001
	40–64 > 85+	<0.001
	65–74 > 75–84	<0.001
	65–74 > 85+	<0.001
	75–84 > 85+	0.048
**Who completed survey ^1^**		
Patient	18–39 > 85+	<0.001
	40–64 > 75–84	<0.001
	40–64 > 85+	<0.001
	65–74 > 75–84	0.004
	65–74 > 85+	<0.001
	75–84 > 85+	<0.001
Patient with help/someone else	18–39 < 85+	<0.001
	40–64 < 75–84	<0.001
	40–64 < 85+	<0.001
	65–74 < 75–84	0.004
	65–74 < 85+	<0.001
	75–84 < 85+	<0.001
**Self-rated health ^2^**		
	18–39 > 75–84	0.011
	18–39 > 85+	<0.001
	40–64 > 85+	0.001
	65–74 > 85+	0.004

Note: Post hoc tests were only conducted for variables for which we found a significant difference across age groups. Only the results of significant post hoc comparisons are presented. ^1^ For nominal variables, only groups that had significant post hoc tests are included in the table; ^2^ for ordinal variables, the groups are not listed because post hoc tests were applied across groups; ^3^ significance values have been adjusted by the Bonferroni correction for multiple tests.

**Table A2:** Post hoc comparisons between age groups for clinical characteristics showing a significant difference across age groups.

	Post Hoc Comparisons	Adj *p ^6^*
**Tumor site ^1^**		
Hematological	40–64 < 65–74	0.037
	40–64 < 75–84	<0.001
	40–64 < 85+	<0.001
	65–74 < 85+	0.005
Breast	18–39 > 65–74	<0.001
	18–39 > 75–84	<0.001
	18–39 > 85+	<0.001
	40–64 > 65–74	<0.001
	40–64 > 75–84	<0.001
	40–64 > 85+	<0.001
Gastrointestinal	40–64 > 85+	0.046
Genitourinary	40–64 < 65–74	<0.001
	40–64 < 75–84	<0.001
Skin ^3^	65–74 < 85+	0.047
	75–84 < 85+	0.015
Central nervous system	18–39 > 40–64	0.005
	18–39 > 65–74	<0.001
	18–39 > 75–84	<0.001
**Time since diagnosis ^2^**		
	18–39 < 40–64	0.019
	18–39 < 65–74	<0.001
	18–39 < 75–84	<0.001
	18–39 < 85+	<0.001
	40–64 < 75–84	<0.001
	40–64 < 85+	<0.001
	65–74 < 85+	<0.001
	75–84 < 85+	0.014
**Type of cancer centre where care was received ^1^**		
Regional	40–64 < 65–74	0.008
	40–64 < 75–84	0.001
**Involvement of family doctor in care while on cancer treatment ^2,4^**
	40–64 < 75–84	0.015
	40–64 < 85+	0.032
**Treatment intent ^1^**		
Cure	18–39 > 40–64	0.005
	18–39 > 65–74	<0.001
	18–39 > 75–84	<0.001
	18–39 > 85+	<0.001
	40–64 > 75–84	0.024
	40–64 > 85+	<0.001
	65–74 > 85+	<0.001
	75–84 > 85+	0.003
Control	18–39 < 40–64	0.014
	18–39 < 65–74	0.001
	18–39 < 75–84	<0.001
	18–39 < 85+	<0.001
	40–64 < 75–84	0.046
	40–64 < 85+	<0.001
	65–74 < 85+	0.009
Unsure	40–64 < 85+	0.021
	65–74 < 85+	0.019
**Type of cancer treatment(s) received (not mutually exclusive) ^1,5^**
IV chemotherapy	18–39 > 85+	0.001
	40–64 > 75–84	< 0.001
	40–64 > 85+	< 0.001
	65–74 > 75–84	0.022
	65–74 > 85+	< 0.001
	75–84 > 85+	0.028
Oral chemotherapy	18–39 < 85+	0.023
	40–64 < 85+	0.017

Note: Post hoc tests were only conducted for variables for which we found a significant difference across age groups. Only results of significant post hoc comparisons are presented. ^1^ For nominal variables, only groups that had significant post hoc tests are included in the table; ^2^ for ordinal variables, the groups are not listed because post hoc tests were applied across groups; ^3^ skin includes melanoma and non-melanoma skin cancers; ^4^ for family doctor involvement, ‘do not have’ and ‘unsure’ were considered not applicable responses and were not included in the post hoc testing; ^5^ based on tumor registry data—some respondents received more than one type of treatment; ^6^ significance values have been adjusted by the Bonferroni correction for multiple tests.
